# Effect of incorporating bone char with sulfur or humic acid on phosphorus availability and spinach growth in calcareous sandy soil

**DOI:** 10.1038/s41598-025-29041-y

**Published:** 2025-12-26

**Authors:** Abdallah M. Barakat, Adel R. A. Usman, Abu El-Eyuoon Abu Zied Amin, Nadia M. K. Roshdy

**Affiliations:** Soils and Water Department, Faculty of Agriculture, Assiut University, Assiut, 71526 Egypt

**Keywords:** Bone char, Humic acid, Modified bone char, Spinach, Sulfur, Sustainable agriculture, Ecology, Ecology, Environmental sciences, Plant sciences

## Abstract

This study investigated the effects of applying modified bone char by sulfur (MBC) with humic acid and co-applying bone char (BC) with sulfur (S) or humic acid (HA) on chemical properties, phosphorus (P) availability, and spinach growth in calcareous sandy soil. This pot experiment has twelve treatments: Control (CK), bone + S (BS), bone + HA (BHA), BC + S (BCS), BC + HA (BCHA), MBC, MBC + HA (MBCHA), acidified BC with 0.1 N H_2_SO_4_ (0.1ABC), acidified BC with 1 N H_2_SO_4_ (1ABC), rock phosphate (RP), RP + S (RPS), and RP + HA (RPHA). The B, BC, MBC, 0.1ABC, 1ABC, and RP were added at 300 mg P kg^− 1^ soil doses. Spinach was grown in this experiment. Applying all treatments significantly increased soil phosphorus availability. Available phosphorus increased from 11.61 mg kg^− 1^ (CK) to 19.70, 19.76, 21.82, 22.25, 22.45, 26.09, 19.58, 21.01, 15.26, 18.95, and 17.77 mg kg^− 1^ for BS, BHA, BCS, BCHA, MBC, MBCHA, 0.1ABC, 1ABC, RP, RPS, and RPHA, respectively. The effectiveness of the treatments in this study on the available phosphorus improvement was in the order of MBCHA > MBC > BCHA > BCS > 1ABC > BHA > BS > 0.1ABC > RPS > RPHA > RP > control. Compared to the control treatment, applying BHA, BCS, BCHA, MBC, MBCHA, 1ABC, RPS, and RPHA to the soil significantly increased the fresh shoot of the spinach plant. Fresh shoot of spinach increased from 46.02 g pot^− 1^ for CK to 54.41, 54.36, 56.94, 50.39, 51.91, 48.83, 54.24, and 49.52 g pot^− 1^ for BHA, BCS, BCHA, MBC, MBCHA, 1ABC, RPS, and RPHA, respectively. The effectiveness of treatments in improving the fresh weight of spinach was in the order of BCHA > BHA ≈ BCS > RPS > MBCHA > MBC > RPHA > 1ABC > control > RP > BS > 0.1ABC. Our results concluded that co-applying bone char with sulfur is optimal for enhancing soil quality indicators and improving fresh and dry shoots of spinach. Due to its cheaper price, it is preferable to add sulfur with bone char rather than humic acid.

## Introduction

Modern agriculture focuses on enhancing the quality and quantity of yields, while also preserving the environment^1^. Spinach (*Spinacea oleracea* L.) is an important leafy vegetable in human nutrition and is cultivated in many places around the world^2^. The spinach plant is low in calories and a main source of vitamins C and A, rich in minerals^3^, a source of total phenolic content, and antioxidant activity, which is considered essential for human health^4^. Spinach is utilized in traditional medicine to treat diabetes, leprosy, asthma, urinary diseases, lung inflammation, joint pains, thirst, sore throat, scabies, vomiting, ringworm, sore eye, cold, sneezing, fever, and diseases related to the brain and heart^5^. Using spinach in the daily diet can lower the risk of cancer^6^.

A common problem in agricultural soils worldwide is phosphorus (P) deficiency, one of the most limiting macronutrients for crop productivity. Where more than 40% of the world’s arable soil suffers from phosphorus deficiency^7^. So, global demand for rock phosphate has increased for the manufacture of phosphate fertilizers to meet the increase in agricultural production in order to meet the food needs resulting from massive population growth^8^. Rock phosphate is expected to be depleted within the next 70 to 140 years without proper management, but in the case of proper phosphorus management, it is possible to postpone depletion for 50 years^9^. Moreover, the excessive use of phosphate fertilizers in agriculture led to an increase in the content of heavy metals and radioactive elements in the soil, which causes the deterioration of ecosystems^10^. Rapid population growth in the world has led to an increase in demand for food, especially an increase in the rate of meat consumption. This, in turn, leads to a significant increase in slaughterhouse wastes (blood, feathers, bone), which are sources of many diseases that affect humans and animals alike^11^. Generally, the production of increasing quantities of by-products poses a major challenge to modern society; their valorization, that is, converting them into valuable compounds with technological applications, is the best way forward, in line with the principles of the circular economy, and represents one of the goals of sustainable development^12^. The utilization of diverse secondary raw materials represents a key step toward achieving a circular phosphorus economy. Phosphorus recycling plays a vital role in sustainable phosphorus management and is fundamental to achieving a circular economy^13^. Bones include huge amounts of inorganic calcium phosphate known as biological apatite^14^. Recycling animal bones in Ethiopia could potentially cover between 28% and 58% of the annual demand for phosphate fertilizers^15^. Converting bones into bone char through a thermal conversion process is considered a promising strategy, a safe and renewable source of phosphate fertilizers that plays an important role in sustainable agriculture^16,17^. Bone char is a solid product produced from the pyrolysis process of de-fatted and de-gelatinized animal bones in the total or partial absence of oxygen at temperatures of 300 to 1050°C^18^. Bone char amendment is considered an alternative source of phosphate fertilizers because it has many properties, such as being rich in phosphorus, a renewable source, and cheap, in addition to avoiding soil contamination with heavy metals and radionuclides, thus playing an important role in sustainable agriculture^19,20^. The main advantages of animal bone char are its abundance, low cost, and purity (no heavy metal content)^20^. Animal bones are rich in phosphate, which can be recovered and converted into bone char —a material with significant fertilizer potential. The use of recycled phosphorus fertilizers, such as bone char, helps close the phosphorus cycle and promotes the development of a circular economy^13^. Nutrient circularity is an effective approach in reducing the consumption of virgin material and maximizing resource efficiency. As a double-edged weapon, these practices need to be implemented with caution^21^.

Phosphorus dissolution from bone char in the soil is dependent on the chemical composition of the soil solution, organic matter, and mineralogical composition of the soil^22^. The water-soluble form of phosphorus in bone char and soil amending with bone char is low^23^. Bone char is a promising fertilizer for recycling wastes rich in phosphorus, but the phosphorus contained in bone char has low solubility, which can be increased by modifying bone char and mixing it with other materials. Therefore, this study hypothesizes that producing bone char rich in sulfur, as well as mixing bone char with sulfur or humic acid, will lead to an increase in the phosphorus release from bone char in calcareous sandy soil and stimulate plant growth. There are few studies on the use of bone char as an alternative to phosphate fertilizers, and most of these studies were conducted in acid soils and were mostly laboratory studies. So, we wanted to evaluate the performance of co-applying sulfur-modified bone char with humic acid and combining bone char with sulfur or humic acid, as well as compare them with phosphate rock on the effect on soil chemical properties, nutrient availability, and the apparent recovery efficiency of phosphorus in a pot experiment by growing spinach plants in calcareous sandy soil. Therefore, the goals of this study were to examine the influence of applying modified bone char with humic acid, as well as co-applying bone char with sulfur or humic acid on soil chemical properties, phosphorus availability, apparent recovery efficiency of phosphorus, and growth of spinach in calcareous sandy soil.

## Materials and methods

### Amendments preparation

Cow bones were collected from a butcher shop in Assiut City, Assiut, Egypt. This is considered waste that will be thrown in the garbage. Then we took the collected bones, removed the fats and gelatin from them, and broke the bones into pieces 10 to 20 cm long. The bones were then crushed into powder by a stainless-steel grinder, which was then passed through a 1 mm diameter sieve. The bone powder alone, or the combination of bone powder with 10% elemental sulfur were pyrolyzed at 380 ± 10 °C for four hours in an outdoor pyrolysis reactor in a stainless-steel cylindrical container with a radius of 30 cm and a length of 60 cm to produce bone char (BC) and modified bone char by sulfur (MBC), respectively. After the pyrolysis process, it turns into bone char. Acidified bone char was produced from treated bone char powder with 0.1 and 1 N H_2_SO_4_, where sulfuric acid was added to the bone char at saturation. Then, the acidified bone char was dried at 105–110 °C for 24 h. The chemical properties of bone, bone char, modified bone char, and acidified bone char are shown in Table 1.

**Table 1:** Some important properties of amendments used in this experiment. Data were average ± standard error (SE).

Amendments	pH_(1:5)_	EC_(1:5)_ (ds m^− 1^)	Olsen-*P* (mg kg ^− 1^)	Total content (g kg ^− 1^)				
				*P*	C	H	*N*	S
B	6.7 ± 0.14	0.87 ± 0.01	446.29 ± 19.70	69.64 ± 0.51	218.1	18.7	42.6	4.4
BC	8.15 ± 0.21	0.91 ± 0.00	627.85 ± 20.48	105.13 ± 3.56	111	18.5	16.1	4.7
MBC	6.95 ± 0.07	0.77 ± 0.02	676.24 ± 5.43	106.63 ± 1.44	150	12.3	23.4	19.6
0.1ABC	6.85 ± 0.07	0.88 ± 0.02	708.95 ± 15.06	127.51 ± 1.67	68.9	6.3	11.8	5.4
1ABC	5.5 ± 0.28	1.41 ± 0.09	770.93 ± 6.05	153.11 ± 7.79	80.3	10.2	14.9	21.5
RP	8.2 ± 0.28	2.30 ± 0.04	22.35 ± 1.49	87.47 ± 0.03	10.2	13.9	0.59	17.5
HA	8.85 ± 0.35	4.54 ± 0.05	-	0.82 ± 0.03	234.7	10.5	8.6	14.2
S	4.55 ± 0.21	0.64 ± 0.01	7.40 ± 0.60	-	3.4	8.5	0.8	957.4

B: bone; BC: bone char; MBC: modified bone char; 0.1ABC: acidified BC with 0.1 N H_2_SO_4_; 1ABC: acidified BC with 1 N H_2_SO_4_; RP: rock phosphate; HA: humic acid; S: sulfur.

## Materials

We brought rock phosphate and sulfur from the superphosphate factory in Assiut, Egypt, as well as humic acid, spinach seeds, and ammonium nitrate fertilizer were purchased from an agricultural supply store. The chemical properties of rock phosphate, sulfur, and humic acid are shown in Table 1.

### Soil preparation

Soil samples for the cultivated layer (0–30 cm) were collected from Al-Gharib Farm, Faculty of Agriculture, Assiut University, Assiut, Egypt. Soil samples were air-dried and ground to pass through a 2 mm sieve. The physical and chemical properties of the soil under study are presented in Table 2. The soil taken was used in the pot experiment under study.

**Table 2:** Some chemical and physical properties of the soil under study. Data were average ± standard error (SE).

Property	Unit	Value
Sand	g kg^− 1^	922 ± 8.48
Silt	g kg^− 1^	62 ± 2.82
Clay	g kg^− 1^	16 ± 5.65
Texture		Sand
pH (1:1)		7.83 ± 0.35
EC (1:1)	dS m^− 1^	0.70 ± 0.05
CaCO_3_	g kg^− 1^	195 ± 4.24
O.M	g kg^− 1^	3.57 ± 0.00
Soluble Ca	mmol kg^− 1^	1.37 ± 0.06
Soluble Mg	mmol kg^− 1^	0.47 ± 0.15
Soluble Na	mmol kg^− 1^	0.71 ± 0.02
Soluble K	mmol kg^− 1^	0.49 ± 0.01
Soluble CO_3_	mmol kg^− 1^	–
Soluble HCO_3_	mmol kg^− 1^	2.47 ± 0.21
Soluble Cl	mmol kg^− 1^	1.53 ± 0.12
Soluble SO_4_	mmol kg^− 1^	0.60 ± 0.07
Available P	mg kg^− 1^	13.90 ± 0.99
Available K	mg kg^− 1^	148.04 ± 8.60

### Design of the pot experiment

Each plastic pot (15 cm depth × 12.5 cm base diameter × 15 cm top diameter) was filled with three kilograms of this soil. This experiment consists of twelve treatments: Control (unamended soil, CK), bone + sulfur (BS), bone + humic acid (BHA), bone char + sulfur (BCS), bone char + humic acid (BCHA), sulfur-modified bone char (MBC), sulfur-modified bone char + humic acid (MBC + HA), acidified bone char with 0.1 N H_2_SO_4_ (0.1ABC), acidified bone char with 1 N H_2_SO_4_ (1ABC), rock phosphate (RP), rock phosphate + sulfur (RPS), rock phosphate + humic acid (RPHA). The experiment was arranged in a completely randomized design with three replications. The B, BC, MBC, 0.1ABC, 1ABC, and RP were added to the soil at doses of 12.9, 8.7, 9.3, 8.7, 8.7, and 10.2 g pot^− 1^, respectively. The doses of these amendments were added based on the total phosphorus at 300 mg P kg^− 1^ soil (equivalent to 720 kg ha^− 1^). However, HA was added at a dose of 0.6 g pot^− 1^ (equivalent to 480 kg ha^− 1^), and S was added at a dose of 1.8 g pot^− 1^ (equivalent to 1440 kg ha^− 1^), respectively. Eight spinach seeds were sown in each pot on 3 November 2022, irrigated with tap water (0.47 dS m^− 1^). After 18 days of sowing, five plants were thinned in each pot. Irrigation was applied according to the growth status of the spinach plants. Each pot was fertilized with 268 mg of nitrogen. Nitrogen fertilizer was applied as a form of ammonium nitrate solution in two doses. The chlorophyll content was measured by SPAD-502, and the plant height of the spinach plant was measured after 55 days from planting. The spinach plant was harvested after 55 days from planting. Then, the fresh shoot of spinach was recorded for each pot. The shoot of spinach was washed with distilled water and oven-dried at 70 °C. Then, the weight of the dry shoot was recorded. Soil samples were taken from each pot after harvesting, air-drying, crushing, and keeping for chemical analysis. This open pot experiment was performed at the Department of Soils and Water, Faculty of Agriculture, Assiut University, Assiut, Egypt.

### Analyses of bone char and soil

The particle size distribution of the soil under study was determined using the pipette method^24^. Soil calcium carbonate content was estimated before the experiment using a calcimeter apparatus^25^. Soil organic matter is estimated by the dichromate oxidation procedure. We took 2 g of air-dried soil and placed it into a 500 mL conical flask, followed by adding 10 mL of 1 N K_2_Cr_2_O_7_ solution, then 20 mL of concentrated H_2_SO_4_ was added. The mixture was mixed thoroughly and allowed to cool. The mixture was diluted in the flask with 200 mL of distilled water, and the contents were agitated in the flask. 2 mL of diphenylamine indicator was added. The solution was titrated with standard FeSO_4_ solution to a brilliant green color^26^. Soil pH was measured in a suspension (1:1). We took 50 g of air-dried soil and transferred it into a 100 mL plastic cup. 50 ml of distilled water was added. This suspension was mixed thoroughly for 1 min using a glass rod, then let stand for 30 min, and stirred 2–3 times during this period. The glass electrode was inserted into the cup so that the tip of the glass electrode reached deep into the soil to measure pH using pH-meter^27^. The electrical conductivity (EC) of soil extracts was measured at a 1:1 extract. A 50 g sample of air-dried soil was taken and placed into a 100 mL plastic cup, followed by the addition of 50 mL of distilled water. This suspension was mixed thoroughly for 1 min using a glass rod, then let stand for 30 min, stirred 2–3 times during this period. Then, the soil suspension was filtered through filter paper. After obtaining the soil extract, the electrical conductivity was measured using an EC-meter^27^. Soluble calcium and magnesium in all soil extracts were estimated by titration with 0.01 N from Na_2_EDTA solution; Soluble K and Na were analyzed by a flame photometer^27^. Soluble sulfate was determined by the turbidimetric method, soluble bicarbonate (HCO_3_) + carbonate (CO_3_) was determined by titration with hydrochloric acid dilution^28^, and soluble chloride was determined using silver nitrate solution^27^. Available nitrogen in soil samples after harvesting spinach plant was extracted by 1 M KCl^29^. A 10 g of air-dried soil was taken and placed in a 100 mL plastic bottle, followed by the addition of 50 mL of 1 N KCl solution. The mixture was shaken for one hour and then filtered through filter paper. The available nitrogen in soil extracts was determined by the Kjeldahl method^30^. Available phosphorus (Olsen-P) in the samples of soil and bone char was extracted by 0.5 M NaHCO_3_ at pH 8.5^31^. 2.5 g of soil was taken and placed in a 100 mL plastic bottle, followed by the addition of 50 mL of 0.5 M NaHCO₃ solution. The mixture was shaken for 30 min and then filtered through filter paper. Phosphorus in the extracts was measured by colorimetric analysis using the chlorostannous phosphomolybdic acid method^27^. Available potassium in soil samples was extracted with 1 M ammonium acetate, pH 7. A 5 g of soil was taken and placed in a 100 mL plastic bottle, followed by the addition of 50 mL of 1 M ammonium acetate, pH 7. The mixture was shaken for 30 min and then filtered through filter paper, and then the potassium in the extracts was measured by a flame photometer^28^. The amendments: B, BC, MBC, 0.1ABC, 1ABC, RP, and HA were digested with concentrated H_2_SO_4_, HNO_3_, and HClO_4_ to determine the total phosphorus^32^. Phosphorus in extracts was measured colorimetrically by the chlorostannous phosphomolybdic acid method in the sulfuric acid system^27^. Total C, H, N, and S were determined in all solid materials, such as B, BC, MBC, 0.1ABC, 1ABC, RP, HA, and S, using a CHNS elemental analyzer (Elementar Vario EL, Germany).

### Plant analysis

Total elements such as nitrogen, phosphorus, potassium, and calcium were determined in dried shoots of spinach plant samples after digestion with a mixture of H_2_SO_4_-H_2_O_2_. A 0.4 g portion of the plant sample was placed in a digestion tube, to which 15 mL of digestion mixture was added. The sample was left to stand overnight before digestion. The digestion was carried out in a closed vessel using a digestion unit. Initially, the temperature was set at 120 °C and then gradually increased to 360 °C, continuing until the digestion was complete and the solution turned clear and colorless^33^. Total nitrogen in all digestive samples was estimated by the Micro-Kjeldahl method, and phosphorus was measured colorimetrically by the phosphomolybdic acid method in a sulfuric acid system^27^. Potassium was analyzed by flame photometry. Calcium and magnesium were determined by titration with Na_2_EDTA solution. Plant nutrient uptake (pot mg ^− 1^) was calculated using the following Equation^34^.

$$\:\mathrm{N}\mathrm{u}\mathrm{t}\mathrm{r}\mathrm{i}\mathrm{e}\mathrm{n}\mathrm{t}\:uptake\:\left(mg\:{pot}^{-1}\right)=\frac{\mathrm{N}\mathrm{u}\mathrm{t}\mathrm{r}\mathrm{i}\mathrm{e}\mathrm{n}\mathrm{t}\:\mathrm{c}\mathrm{o}\mathrm{n}\mathrm{c}\mathrm{e}\mathrm{n}\mathrm{t}\mathrm{r}\mathrm{a}\mathrm{t}\mathrm{i}\mathrm{o}\mathrm{n}\:\mathrm{i}\mathrm{n}\:\mathrm{d}\mathrm{r}\mathrm{y}\:\mathrm{s}\mathrm{h}\mathrm{o}\mathrm{o}\mathrm{t}\:\left(\mathrm{m}\mathrm{g}\:{kg}^{-1}\right)\:\mathrm{X}\:\mathrm{d}\mathrm{r}\mathrm{y}\:\mathrm{s}\mathrm{h}\mathrm{o}\mathrm{o}\mathrm{t}\:\left(\mathrm{g}\:{pot}^{-1}\right)}{1000}$$

The apparent recovery efficiency of phosphorus by the spinach plant was calculated using the following Equation^35^.

$$\:\mathrm{A}\mathrm{p}\mathrm{p}\mathrm{a}\mathrm{r}\mathrm{e}\mathrm{n}\mathrm{t}\:\mathrm{r}\mathrm{e}\mathrm{c}\mathrm{o}\mathrm{v}\mathrm{e}\mathrm{r}\mathrm{y}\:\mathrm{e}\mathrm{f}\mathrm{f}\mathrm{i}\mathrm{c}\mathrm{i}\mathrm{e}\mathrm{n}\mathrm{c}\mathrm{y}\:\mathrm{o}\mathrm{f}\:\mathrm{p}\mathrm{h}\mathrm{o}\mathrm{s}\mathrm{p}\mathrm{h}\mathrm{o}\mathrm{r}\mathrm{u}\mathrm{s}\:\left(\%\right)=\frac{(\mathrm{p}\mathrm{h}\mathrm{o}\mathrm{s}\mathrm{p}\mathrm{h}\mathrm{o}\mathrm{r}\mathrm{u}\mathrm{s}\:\mathrm{u}\mathrm{p}\mathrm{t}\mathrm{a}\mathrm{k}\mathrm{e}\:\mathrm{i}\mathrm{n}\:\mathrm{f}\mathrm{e}\mathrm{r}\mathrm{t}\mathrm{i}\mathrm{l}\mathrm{i}\mathrm{z}\mathrm{e}\mathrm{d}\:\mathrm{p}\mathrm{o}\mathrm{t}-\mathrm{p}\mathrm{h}\mathrm{o}\mathrm{s}\mathrm{p}\mathrm{h}\mathrm{o}\mathrm{r}\mathrm{u}\mathrm{s}\:\mathrm{u}\mathrm{p}\mathrm{t}\mathrm{a}\mathrm{k}\mathrm{e}\:\mathrm{i}\mathrm{n}\:\mathrm{u}\mathrm{n}\mathrm{f}\mathrm{e}\mathrm{r}\mathrm{t}\mathrm{i}\mathrm{l}\mathrm{i}\mathrm{z}\mathrm{e}\mathrm{t}\:\mathrm{p}\mathrm{o}\mathrm{t})}{Quantity\:of\:phosphorus\:applied\:per\:pot}$$

The phosphorus budget was calculated using the following Equation^36,37^.

$$\:Phosphorus\:budget=\left(total\:phosphorus\:input\right)-\left(total\:phosphorus\:output\right)$$

Total P input includes available phosphorus in the soil before cultivation and the amount of phosphorus applied.

Total P output includes available phosphorus in the soil after harvesting and total phosphorus uptake by the spinach plant.

### Statistical analysis

The collected data were statistically analyzed using the SAS program (SAS Institute, Inc., NC, USA, version 9.00). The analysis of variance (ANOVA) was performed on the data. Each variable was reported as the mean of three replicates for one treatment. Significant differences among treatments were carried out by Tukey’s honestly significant difference test (Tukey’s HSD) at the 0.01 level of probability (p), which corresponds to a 99% confidence level.

## Results

### Effect of amendments on soil chemical properties

**Fig. 1:**
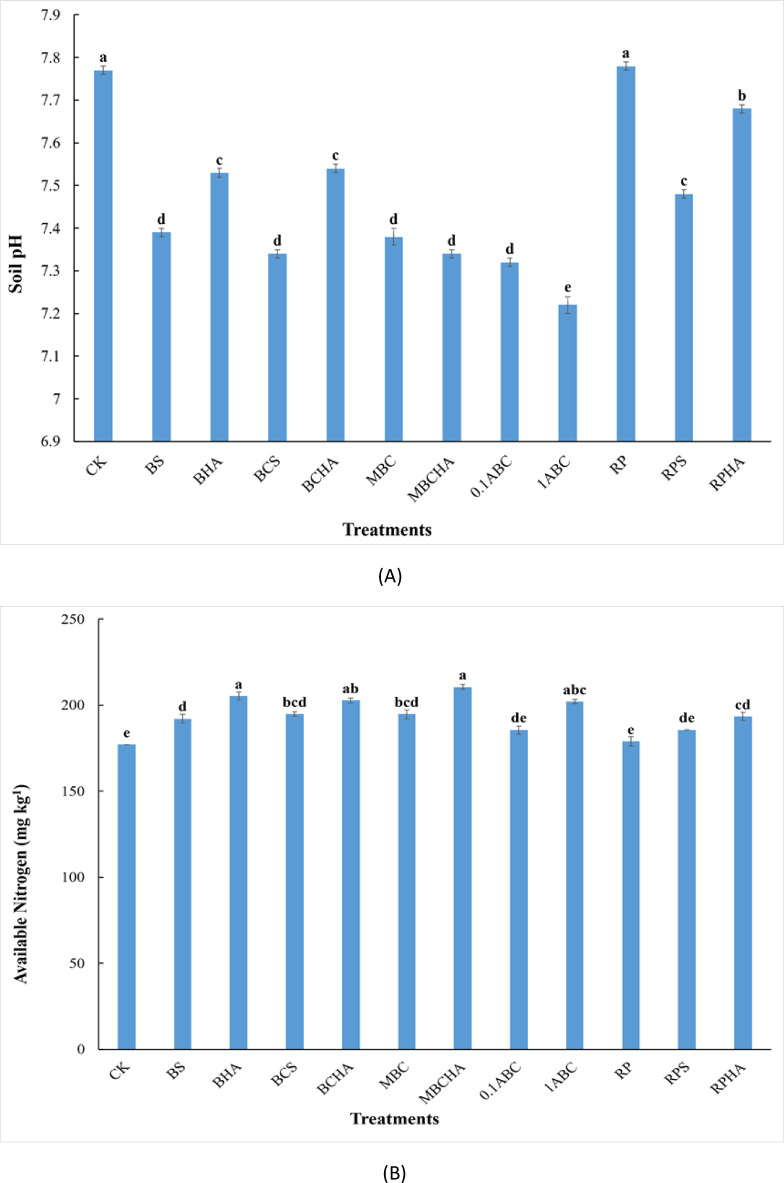
Changes in pH and available nitrogen in calcareous sandy soil as affected by different amendments after the harvesting of spinach. Each value indicates the average of three replicates, with the standard error shown by the vertical bars. Different lowercase letters on each bar indicate the significant differences among treatments by using Tukey’s Honestly Significant Difference test at *p* ≤ 0.01 (corresponds to a 99% confidence level). CK: control (unamended soil); BS: bone + sulfur; BHA: bone + humic acid; BCS: bone char + sulfur; BCHA: bone char + humic acid; MBC: modified bone char; MBCHA: modified bone char + humic acid; 0.1ABC: acidified bone char with 0.1 N H_2_SO_4_; 1ABC: acidified bone char with 1 N H_2_SO_4_; RP: rock phosphate; RPS: rock phosphate + sulfur; RPHA: rock phosphate + humic acid.

The application of BS, BHA, BCS, BCHA, MBC, MBCHA, 0.1ABC, 1ABC, RPS, and RPHA to calcareous sandy soil caused a significant decrease (*P* ≤ 0.01) in pH compared to the control treatment after the harvesting of spinach (Fig. 1A). Soil pH values decreased from 7.77 for the control treatment to 7.39, 7.53, 7.34, 7.54, 7.38, 7.34, 7.32, 7.22, 7.78, 7.48, and 7.68 for the treatments BS, BHA, BCS, BCHA, MBC, MBCHA, 0.1ABC, 1ABC, RP, RPS, and RPHA respectively. The lowest value of soil pH was observed when adding the 1ABC treatment to the soil under study (Fig. 1A).

The applications of all treatments in the soil did not show any significant differences in the electrical conductivity after the harvesting of the spinach plant (Table 3). Compared to the control treatment, the application of BS, BCS, BCHA, MBC, and MBCHA treatments significantly reduced soluble sodium in calcareous sandy soil (Table 3). The concentrations of soluble sodium in the soil extract decreased from 0.41 mmol kg^− 1^ soil for the control treatment to 0.36, 0.37, 0.36, 0.35, 0.36, 0.34, 0.38, 0.38, 0.37, and 0.38 mmol kg^− 1^ soil for BS, BHA, BCS, BCHA, MBC, MBCHA, 0.1ABC, 1ABC, RPS, and RPHA treatments, respectively. The lowest value of soluble sodium in the soil extract was observed in the application of MBCHA. Applying BS, BCS, BCHA, MBC, and MBCHA significantly increased soluble potassium in calcareous sandy soil (Table 3). Where soluble potassium increased from 0.24 mmol kg^− 1^ soil for the control treatment to 0.29, 0.31, 0.28, 0.29, and 0.31 mmol kg^− 1^ soil for BS, BCS, BCHA, MBC, and MBCHA treatments, respectively. The highest value of soluble potassium was observed in the application of the MBCHA treatment (Table 3). Compared with the control treatment, the addition of BS, BHA, BCS, BCHA, MBC, MBCHA, 0.1ABC, and 1ABC, RP, RPS, and RPHA significantly increased soluble calcium + magnesium in the soil under study. Where soluble Ca + Mg increased from 1.00 mmol kg^− 1^ soil for control treatment to 1.66, 1.50, 2.00, 2.00, 2.00, 1.33, 2.20, 1.26, 1.23, and 1.10 mmol kg^− 1^ soil for BS, BHA, BCS, BCHA, MBC, MBCHA, 0.1 ABC, 1ABC, RP, RPS, and RPHA, respectively. The highest concentration of soluble Ca + Mg was observed in the 1ABC application (Table 3). The application of BS, BCS, MBC, MBCHA, 0.1ABC, 1ABC, and RPS treatments to calcareous sandy soil resulted in a significant increase in soluble sulfate compared with the control treatment (Table 3). The concentration of soluble sulfate increased from 0.29 mmol kg^− 1^ for the control treatment to 0.48, 0.38, 0.50, 0.37, 0.49, 0.47, 0.51, 0.58, 0,36, 0.49, and 0.35 mmol kg^− 1^ for BS, BHA, BCS, BCHA, MBC, MBCHA, 0.1 ABC, 1ABC, RP, RPS, and RPHA treatments, respectively. The highest concentration of soluble sulfate was observed when 1ABC was applied (Table 3). Compared to the control treatment, BS, BHA, BCS, BCHA, MBC, and MBCHA applications significantly increased soluble bicarbonate in calcareous sandy soil. But the addition of 0.1ABC, 1ABC, and RPS resulted in a significant decrease in soluble bicarbonate. Where the concentration of soluble bicarbonate increased from 2.06 mmol kg^− 1^ soil for the control treatment to 2.76, 2.56, 2.56, 2.80, 2.96, 2.90, and 2.26 mmol kg^− 1^ for soil BS, BHA, BCS, BCHA, MBC, MBCHA, and RPHA treatments, respectively. The highest value of soluble bicarbonate was observed in the MBC treatment.

**Table 3:** Effect of amendments used in this experiment on electrical conductivity, soluble cations, and soluble anions in calcareous sandy soil. Values displayed are averages ± standard error (*n* = 3 analytical replicates).

Treatment	EC dS m^− 1^	Concentration (mmol kg^− 1^)					
		Na	K	Ca + Mg	HCO_3_	Cl	SO4
CK	0.56 ± 0.06a	0.41 ± 0.01ab	0.24 ± 0.01d	1.00 ± 0.05f	2.06 ± 0.03 cd	1.13 ± 0.07abc	0.29 ± 0.02c
B + S	0.60 ± 0.03a	0.36 ± 0.01bc	0.29 ± 0.00ab	1.66 ± 0.03b	2.76 ± 0.07a	0.93 ± 0.07c	0.48 ± 0.02ab
B + HA	0.66 ± 0.09a	0.37 ± 0.01ac	0.26 ± 0.00bd	1.50 ± 0.05bcd	2.56 ± 0.07ab	1.13 ± 0.07abc	0.38 ± 0.04bc
BC + S	0.63 ± 0.06a	0.36 ± 0.02c	0.31 ± 0.01a	2.00 ± 0.05a	2.56 ± 0.07ab	1.26 ± 0.07abc	0.50 ± 0.03ab
BC + HA	0.70 ± 0.03a	0.35 ± 0.01c	0.28 ± 0.00bc	1.56 ± 0.06bc	2.80 ± 0.10ab	1.46 ± 0.07ab	0.37 ± 0.03bc
MBC	0.66 ± 0.00a	0.36 ± 0.01c	0.29 ± 0.00ab	2.00 ± 0.03a	2.96 ± 0.09a	1.26 ± 0.18abc	0.49 ± 0.05ab
MBC + HA	0.63 ± 0.07a	0.34 ± 0.01c	0.31 ± 0.00a	2.00 ± 0.08a	2.90 ± 0.06a	1.06 ± 0.07bc	0.47 ± 0.02ab
0.1ABC	0.70 ± 0.06a	0.38 ± 0.00ac	0.24 ± 0.01d	1.33 ± 0.05cde	1.53 ± 0.07e	1.60 ± 0.20a	0.51 ± 0.02ab
1ABC	0.66 ± 0.07a	0.38 ± 0.01ac	0.24 ± 0.01d	2.20 ± 0.05a	1.73 ± 0.13de	1.53 ± 0.13ab	0.58 ± 0.05a
RP	0.70 ± 0.03a	0.42 ± 0.01a	0.24 ± 0.00d	1.26 ± 0.05cdef	2.06 ± 0.09 cd	1.26 ± 0.07abc	0.36 ± 0.05bc
RP + S	0.660.09a	0.37 ± 0.01abc	0.25 ± 0.00 cd	1.23 ± 0.09def	1.56 ± 0.03e	1.46 ± 0.07ab	0.49 ± 0.04ab
RP + HA	0.73 ± 0.03a	0.38 ± 0.01ac	0.24 ± 0.01d	1.10 ± 0.05ef	2.26 ± 0.12bc	1.46 ± 0.07ab	0.35 ± 0.05bc

Different lowercase letters in each column showed significant differences between treatments by Tukey’s Honestly Significant Difference test at *p* ≤ 0.01 (corresponds to a 99% confidence). CK: control (unnamed soil); BS: bone + sulfur; BHA: bone + humic acid; BCS: bone char + sulfur; BCHA: bone char + humic acid; MBC: modified bone char; MBCHA: modified bone char + humic acid; 0.1ABC: acidified bone char with 0.1 N H_2_SO_4_; 1ABC: acidified bone char with 1 N H_2_SO_4_; RP: rock phosphate; RPS: rock phosphate + sulfur; RPHA: rock phosphate + humic acid. EC: electrical conductivity.

### Effect of amendments on nutrient availability

**Fig. 2:**
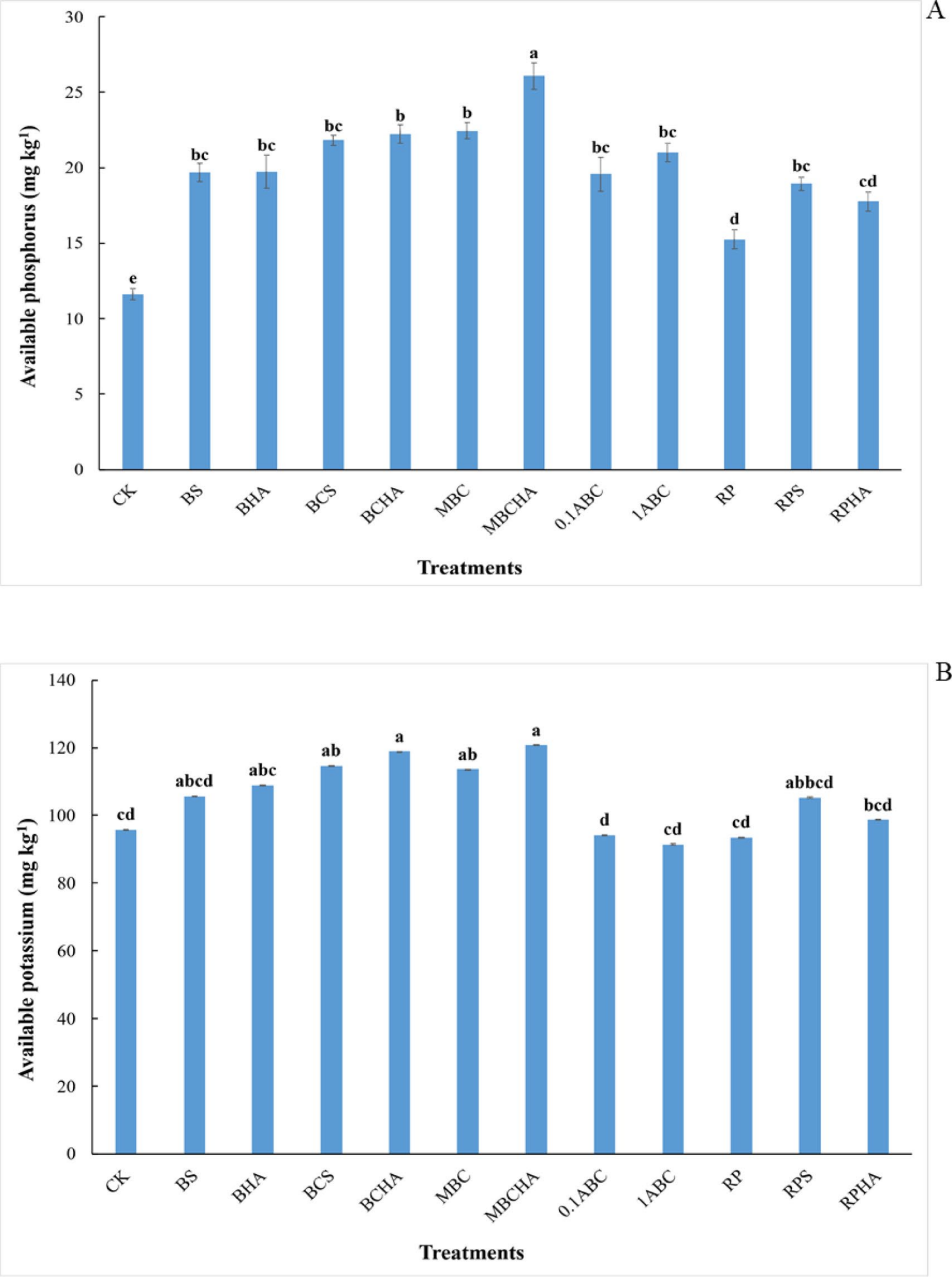
Changes of available phosphorus and potassium in calcareous sandy soil as affected by different amendments after the harvesting of spinach. Each value indicates the average of three replicates, with the standard error shown by the vertical bars. Different lowercase letters on each bar indicate the significant differences among treatments by using Tukey’s Honestly Significant Difference test at *P* < 0.01 (corresponds to a 99% confidence level). CK: control (unamended soil); BS: bone + sulfur; BHA: bone + humic acid; BCS: bone char + sulfur; BCHA: bone char + humic acid; MBC: modified bone char; MBCHA: modified bone char + humic acid; 0.1ABC: acidified bone char with 0.1 N H_2_SO_4_; 1ABC: acidified bone char with 1 N H_2_SO_4_; RP: rock phosphate; RPS: rock phosphate + sulfur; RPHA: rock phosphate + humic acid.

The addition of BS, BHA, BCS, BCHA, MBC, MBCHA, 1ABC, and RPHA to the calcareous sandy soil significantly increased nitrogen availability after the harvesting of the spinach plant compared to the control treatment (Fig. 1B). The concentration of available N increased from 177.00 mg kg^− 1^ soil for the control treatment to 192.13, 205.29, 194.76, 202.66, 194.76, 210.56, 185.55, 202.00, 178.97, 185.55, and 193.45 mg kg^− 1^ soil for BS, BHA, BCS, BCHA, MBC, MBCHA, 0.1ABC, 1ABC, RP, RPS, and RPHA, respectively. The highest value of available nitrogen was observed at applying the MBCHA treatment. The effectiveness of the treatments in this study on improving soil available nitrogen was in the order of MBCHA > BHA > BCHA > 1ABC > BCS > MBC > RPHA > BS > RPS > 0.1ABC > RP > control (Fig. 1B). After the harvesting of the spinach plant, the applications of all treatments in calcareous sandy soil improved significantly phosphorous availability (Fig. 2A). The concentration of available phosphorus increased from 11.61 mg kg^− 1^ soil (control treatment) to 19.70, 19.76, 21.82, 22.25, 22.45, 26.09, 19.58, 21.01, 15.26, 18.95 and 17.77 mg kg^− 1^ soil for BS, BHA, BCS, BCHA, MBC, MBCHA, 0.1ABC, 1ABC, RP, RPS and RPHA treatments, respectively (Fig. 2A). The highest concentration of available phosphorus was observed in calcareous sandy soil with applying MBCHA treatment. The effectiveness of the treatments in this study on the available phosphorus improvement was in the order of MBCHA > MBC > BCHA > BCS > 1ABC > BHA > BS > 0.1ABC > RPS > RPHA > RP > control (Fig. 2). Adding BCS, BCHA, MBC, and MBCHA to calcareous sandy soil led to a significant increase in potassium availability (Table 4). The concentration of available K increased from 95.80 mg kg^− 1^ soil for the control treatment to 105.73, 108.94, 114.71, 118.87, 113.59,120.80, 105.26, and 98.68 mg kg^− 1^ soil for BS, BHA, BCS, BCHA, MBC, MBCHA, RPS, and RPHA treatments, respectively. The highest value of available potassium in calcareous sandy soil was noticed in the MBCHA treatment. The effectiveness of the treatments in this study on the available potassium improvement was in the order of MBCHA > BCHA > BCS > MBC > BHA > BS > RPS > RPHA > control > 0.1ABC > RP > 1ABC (Fig. 2B).

**Table 4:** Effect of the amendments used in this experiment on the height and chlorophyll values of spinach plants as well as phosphorus (P) budget in calcareous sandy soil. Values shown are averages ± standard error (*n* = 3 analytical replicates).

Treatment	Plant height (cm)	Chlorophyll (SPAD)	Total input *P* (mg pot^− 1^)	Total output *P* (mg pot^− 1^)	*P* budget (mg pot^− 1^)
CK	11.36 ± 0.21bc	45.86 ± 1.07bcde	41.7	37.05	4.65 ± 1.17f
BS	12.56 ± 0.40ab	47.46 ± 0.24abcde	941.7	68.41	873.29 ± 1.91bcd
BHA	12.43 ± 0.39ab	45.70 ± 0.35cde	941.7	67.29	874.40 ± 2.60bcd
BCS	13.330.34a	50.13 ± 1.03ab	941.7	74.47	867.23 ± 2.36d
BCHA	13.16 ± 0.11ab	51.26 ± 0.13a	941.7	74.52	867.17 ± 1.90d
MBC	13.56 ± 0.32a	50.00 ± 0.11abc	941.7	76.44	865.26 ± 2.17d
MBCHA	12.96 ± 0.33ab	48.86 ± 1.22abcd	941.7	88.84	852.85 ± 3.58e
0.1ABC	10.23 ± 0.68c	40.86 ± 0.79f	941.7	65.45	876.25 ± 3.32bcd
1ABC	12.56 ± 0.39ab	44.10 ± 0.49ef	941.7	71.65	870.05 ± 2.16 cd
RP	11.36 ± 0.41bc	45.06 ± 0.16def	941.7	48.23	893.47 ± 1.37a
RPS	12.13 ± 0.08ab	47.73 ± 1.93abcde	941.7	61.46	880.23 ± 1.49bc
RPHA	11.40 ± 0.23bc	49.50 ± 0.24abc	941.7	58.32	883.38 ± 1.80ab

Different lowercase letters in each column showed significant differences between treatments by Tukey’s Honestly Significant Difference test at *p* ≤ 0.01 (corresponds to a 99% confidence level). CK: control (unnamed soil); BS: bone + sulfur; BHA: bone + humic acid; BCS: bone char + sulfur; BCHA: bone char + humic acid; MBC: modified bone char; MBCHA: modified bone char + humic acid; 0.1ABC: acidified bone char with 0.1 N H_2_SO_4_; 1ABC: acidified bone char with 1 N H_2_SO_4_; RP: rock phosphate; RPS: rock phosphate + sulfur; RPHA: rock phosphate + humic acid.

### Effect of amendments on the growth parameters of the spinach plant

**Fig. 3:**
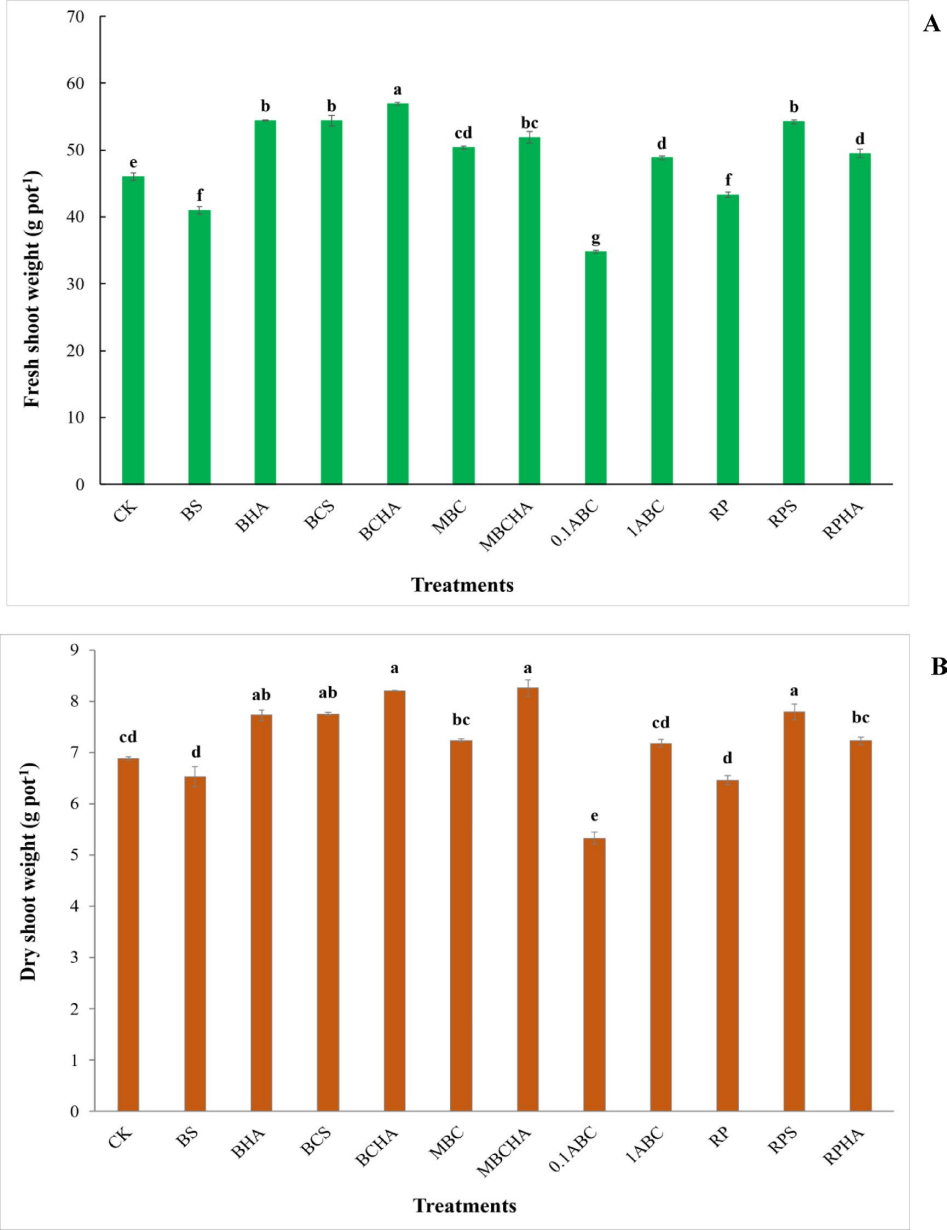
Changes in fresh and dry weight of spinach plant grown in calcareous sandy soil as affected by different amendments. Each value indicates the average of three replicates, with the standard error shown by the vertical bars. Different lowercase letters on each bar indicate the significant differences among treatments by using Tukey’s Honestly Significant Difference test at P < 0.01 (corresponds to a 99% confidence level). CK: control (unamended soil); B+S: bone + sulfur; BHA: bone + humic acid; BCS: bone char + sulfur; BCHA: bone char + humic acid; MBC: modified bone char; MBCHA: modified bone char + humic acid; 0.1ABC: acidified bone char with 0.1 N H2SO4; 1ABC: acidified bone char with 1 N H2SO4; RP: rock phosphate; RPS: rock phosphate + sulfur; RPHA: rock phosphate + humic acid.

The addition of BC + HA significantly increased the chlorophyll value of spinach plant grown in sandy calcareous soil, but the addition of 0.1ABC resulted in a significant decrease in the chlorophyll of spinach compared to the control, treatment (Table 4). The chlorophyll value increased from 45.86 SPAD (control treatment) to 47.46, 50.1, 51.13, 48.86, 47.73, and 49.50 SPAD for the BS, BCS, BCHA, MBC, MBCHA, RPS, and RPHA treatments, respectively. However, the application of BCS and MBC led to a significant increase in the height of the spinach plant compared to the control treatment (Table 4). The height of spinach plants increased from 11.36 cm for the control treatment to 12.56, 12.43, 13.33, 13.16, 13.56, 12.96, 12.56, and 12.13 cm for BS, BHA, BCS, BCHA, MBC, MBCHA, 1ABC, and RPS treatments, respectively. The highest value of the height of the spinach plant was observed at MBC treatment (Table 4). Compared with the control treatment, the applications of BHA, BCS, BCHA, MBC, MBCHA, 1ABC, RPS, and RPHA soil significantly increased the fresh shoot weight of spinach plant grown in the calcareous sandy soil (Fig. 3A). However, the applications of B + S, 0.1ABC, and RP caused a significant decrease in the fresh shoot weight of spinach plant. The fresh shoot weight of the spinach plant increased from 46.02 g pot^− 1^ for the control treatment to 54.41, 54.36, 56.94, 50.39, 51.91, 48.83, 54.24, and 49.52 g pot^− 1^ for BHA, BCS, BCHA, MBC, MBCHA, 1ABC, RPS, and RPHA, respectively. The highest value of the fresh weight of the spinach plant was observed at the BC + HA application (Fig. 3A), while the lowest fresh weight of spinach in this study was recorded for the 0.1ABC treatment. The effectiveness of treatments in improving the fresh weight of spinach was in the order of BCHA > BHA ≈ BCS > RPS > MBCHA > MBC > RPHA > 1ABC > control > RP > BS > 0.1ABC (Fig. 3A). Applying BHA, BCS, BCHA, MBCHA, and RPS caused a significant increase in the dry shoot weight of the spinach plant (Fig. 3B). On the other hand, the addition of 0.1ABC to calcareous sandy soil significantly decreased the dry shoot weight of the spinach plant. The dry weight of the spinach plant increased from 6.88 g pot^− 1^ (control treatment) to 7.73, 7.75, 8.20, 7.24, 8.26, 7.18, 7.79, and 7.23 g pot-1 for BHA, BCS, BCHA, MBC, MBCHA, 1ABC, RPS, and RPHA treatments, respectively. The highest values of dry weight of spinach plant were observed at the BCHA and MBCHA applications (Fig. 3B), while the lowest dry weight of spinach in this study was recorded for the 0.1ABC treatment. Treatments used in this soil showed improvements in the dry shoot weight of the spinach plant in the order of MBCHA > BCHA > RPS > BCS > BHA > MBC > RPHA > 1ABC > CK > BS > RP > 0.1ABC (Fig. 3B).

**Table 5:** Effect of amendments used in this experiment on nutrient concentrations in spinach plant grown in calcareous sandy soil.

Treatments	Nutrient concentrations (g kg^− 1^)			
	*N*	*P*	K	Ca
CK	3.44 ± 0.15e	1.10 ± 0.05 g	36.02 ± 0.82d	98.52 ± 1.93e
B + S	5.13 ± 0.12a	2.27 ± 0.11cde	43.73 ± 0.73a	104.09 ± 1.11cde
B + HA	4.26 ± 0.06bcd	2.53 ± 0.13bcd	38.97 ± 1.07bcd	107.43 ± 1.47bc
BC + S	4.72 ± 0.10ab	2.73 ± 0.12bc	45.19 ± 1.07a	111.32 ± 0.56ab
BC + HA	3.67 ± 0.10bcd	2.62 ± 0.15bcd	42.86 ± 0.47ab	108.54 ± 0.96bc
MBC	4.49 ± 0.06abc	3.08 ± 0.21b	44.57 ± 1.10a	112.99 ± 0.56ab
MBC + HA	4.02 ± 0.10cde	3.86 ± 0.12a	45.04 ± 0.76a	115.78 ± 1.11a
0.1ABC	4.20 ± 0.18bcd	2.42 ± 0.10 cd	40.86 ± 0.72abc	107.98 ± 1.11bc
1ABC	4.55 ± 0.10abc	2.80 ± 0.09bc	43.48 ± 0.79ab	112.43 ± 0.56ab
RP	3.50 ± 0.20e	1.42 ± 0.11 fg	37.33 ± 0.80 cd	99.08 ± 1.11de
RP + S	3.97 ± 0.06cde	1.99 ± 0.16def	40.57 ± 1.33abcd	104.64 ± 1.11 cd
RP + HA	3.68 ± 0.20de	1.72 ± 0.08efg	38.17 ± 1.00 cd	104.09 ± 1.11cde

Values displayed are averages ± standard error (*n* = 3 analytical replicates).

Different lowercase letters in each column showed significant differences between treatments by Tukey’s Honestly Significant Difference test at *p* ≤ 0.01 (corresponds to a 99% confidence level). CK: control (unnamed soil); BS: bone + sulfur; BHA: bone + humic acid; BCS: bone char + sulfur; BCHA: bone char + humic acid; MBC: modified bone char; MBCHA: modified bone char + humic acid; 0.1ABC: acidified bone char with 0.1 N H_2_SO_4_; 1ABC: acidified bone char with 1 N H_2_SO_4_; RP: rock phosphate; RPS: rock phosphate + sulfur; RPHA: rock phosphate + humic acid.

### Effect of amendments on the concentrations of nutrients in the spinach plant

The concentration of nitrogen in the spinach plant increased significantly with applications of BS, BHA, BCS, BCHA, MBC, 0.1ABC, and 1ABC treatments to calcareous sandy soil compared to the control treatment (Table 5). Nitrogen content in spinach plant increased from 3.44 g kg^− 1^ for control treatment to 5.13, 4.26, 4.72, 3.67, 4.49, 4.02, 4.20, 4.55, 3.50, 3.97, and 3.68 g kg^− 1^ for BS, BHA, BCS, BCHA, MBC, MBCHA, 0.1ABC, 1ABC, RP, RPS, and RPHA treatments, respectively. Effectiveness of the treatments in this study on improving nitrogen content in spinach plant was in the order of BS > BCS > 1ABC > MBC > BHA > 0.1ABC > MBCHA > RPS > RPHA > BCHA > RP > CK. The applications of BS, BHA, BCS, BCHA, MBC, MBCHA, 0.1ABC, 1ABC, and RPS to calcareous sandy soil increased significantly phosphorus content in spinach plant in comparison with the control treatment (Table 5). Phosphorus content in spinach plant increased from 1.10 g kg^− 1^ for the control treatment to 2.27, 2.53, 2.73, 2.62, 3.08, 3.86, 2.42, 2.80, 1.42, 1.99, and 1.72 g kg^− 1^ for BS, BHA, BCS, BCHA, MBC, MBCHA, 0.1ABC, 1ABC, RP, RPS, and RPHA, respectively. The highest value of phosphorus content in the spinach plant was observed in the MBCHA treatment (Table 5). Therefore, all treatments increased the phosphorus content in the spinach plant in the order of MBCHA > MBC > 1ABC > BCS > BCHA > BHA > 0.1ABC > RPS > RPHA > RP > CK. The potassium content in the spinach plant increased significantly with the application of BS, BCS, BCHA, MBC, MBCHA, 0.1ABC, and 1ABC to calcareous sandy soil (Table 5). Potassium content in spinach plant increased from 36.02 g kg^− 1^ (control treatment) to 43.73, 38.97, 45.19, 42.86, 44.57, 45.04, 40.86, 43.48, 37.33, 40.57, and 38.17 g kg^− 1^ for BS, BHA, BCS, BCHA, MBC, MBCHA, 0.1ABC, 1ABC, RP, RPS, and RPHA treatments, respectively. The highest value of potassium concentration was observed in the spinach plant under adding the BCS treatment. Compared with the control treatment, the addition of BHA, BCS, BCHA, MBC, MBCHA, 0.1ABC, 1ABC, and RPS to calcareous sandy soil significantly increased calcium concentration in the spinach plant. Calcium concentration increased from 98.52 g kg^− 1^ (control treatment) to 104.09, 107.43, 111.32, 108.54, 112.99, 115.78, 107.98, 112.43, 99.08, 104.64, and 104.09 g kg^− 1^ for BS, BHA, BCS, BCHA, MBC, MBCHA, 0.1ABC, 1ABC, RP, RPS, and RPHA treatments, respectively. The highest calcium content was noticed in the spinach plant after adding the MBCHA treatment (Table 5).

### Effect of amendments on nutrient uptake by the spinach plant

**Fig. 4:**
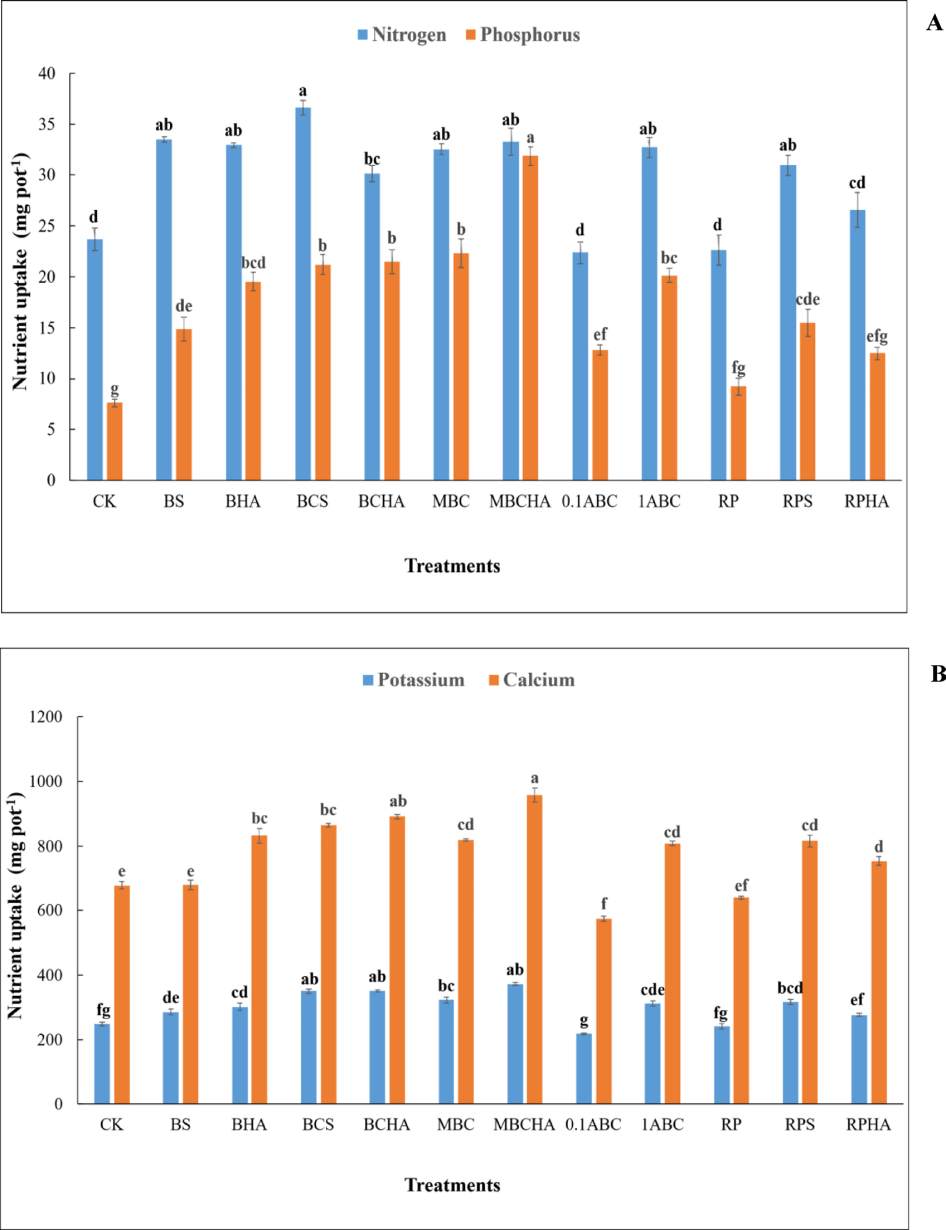
Changes in uptake content of nitrogen and phosphorus, as well as potassium and calcium by spinach plant grown in calcareous sandy soil as affected by different amendments. Each value indicates the average of three replicates, with the standard error shown by the vertical bars. Different lowercase letters on each bar indicate the significant differences among treatments by using Tukey’s Honestly Significant Difference test at *P* < 0.01 (corresponds to a 99% confidence level). CK: control (unamended soil); B + S: bone + sulfur; B + HA: bone + humic acid; BC + S: bone char + sulfur; BC + HA: bone char + humic acid; MBC: modified bone char; MBC + HA: modified bone char + humic acid; 0.1ABC: acidified bone char with 0.1 N H_2_SO_4_; 1ABC: acidified bone char with 1 N H_2_SO_4_; RP: rock phosphate; RP + S: rock phosphate + sulfur; RP + HA: rock phosphate + humic acid.

Nitrogen uptake by spinach plant increased significantly under the applications of BS, BHA, BCS, BCHA, MBC, MBCHA, 1ABC, and RPS treatments to calcareous sandy soil compared to the control treatment (Fig. 4A). Nitrogen uptake content increased from 23.69 mg pot^− 1^ for the control treatment to 33.48, 32.92, 36.63, 30.14, 32.53, 33.27, 32.69, 30.94, and 26.60 mg pot^− 1^ for BS, BHA, BCS, BCHA, MBC, MBCHA, 1ABC, RPS, and RPHA treatments, respectively. The highest content of nitrogen uptake by the spinach plant was noticed after adding BCS treatment (Fig. 4A). The applications of BS, BHA, BCS, BCHA, MBC, MBCHA, 0.1ABC, 1ABC, and RPS treatments to calcareous sandy soil improved significantly phosphorus uptake by spinach compared to the control treatment (Fig. 4A). Phosphorus uptake content increased from 7.62 mg pot^− 1^ for control treatment to 14.88, 19.53, 21.20, 21.47, 22.29, 31.86, 12.84, 20.14, 9.21, 15.49, and 12.48 mg pot^− 1^ for BS, BHA, BCS, BCHA, MBC, MBCHA, 0.1ABC, 1ABC, RP, RPS, and RPHA treatments, respectively (Fig. 4A). The highest phosphorus uptake content by the spinach plant was noticed after adding MBCHA treatment. Compared with the control treatment, the applications of BS, BHA, BCS, BCHA, MBC, MBCHA, 1ABC, and RPS treatments to calcareous sandy soil improved significantly potassium uptake by spinach (Fig. 4B). Potassium uptake content increased from 247.94 mg pot^− 1^ for control treatment to 285.57, 301.49, 350.31, 351.61, 322.91, 372.04, 312.43, 316.06, and 275.97 mg pot^− 1^ for BS, BHA, BCS, BCHA, MBC, MBCHA, 1ABC, RPS, and RPHA treatments, respectively (Fig. 4B). The highest content of potassium uptake by the spinach plant was noticed after adding MBCHA treatment. The applications of BHA, BCS, BCHA, MBC, MBCHA, 1ABC, RPS, and RPHA treatments to calcareous sandy soil significantly improved calcium uptake by the spinach plant compared to the control treatment (Fig. 4B). Calcium uptake increased from 678.11 mg pot^− 1^ for the control treatment to 679.33, 831.09, 863.15, 890.41, 818.46, 956.73, 807.65, 815.91, and 753.05 mg pot^− 1^ for BS, BHA, BCS, BCHA, MBC, MBCHA, 1ABC, RPS, and RPHA treatments, respectively. The highest calcium uptake content by the spinach plant was noticed after adding MBC + HA treatment (Fig. 4B).

### Effect of amendments on apparent recovery efficiency and the budget of phosphorus

**Fig. 5:**
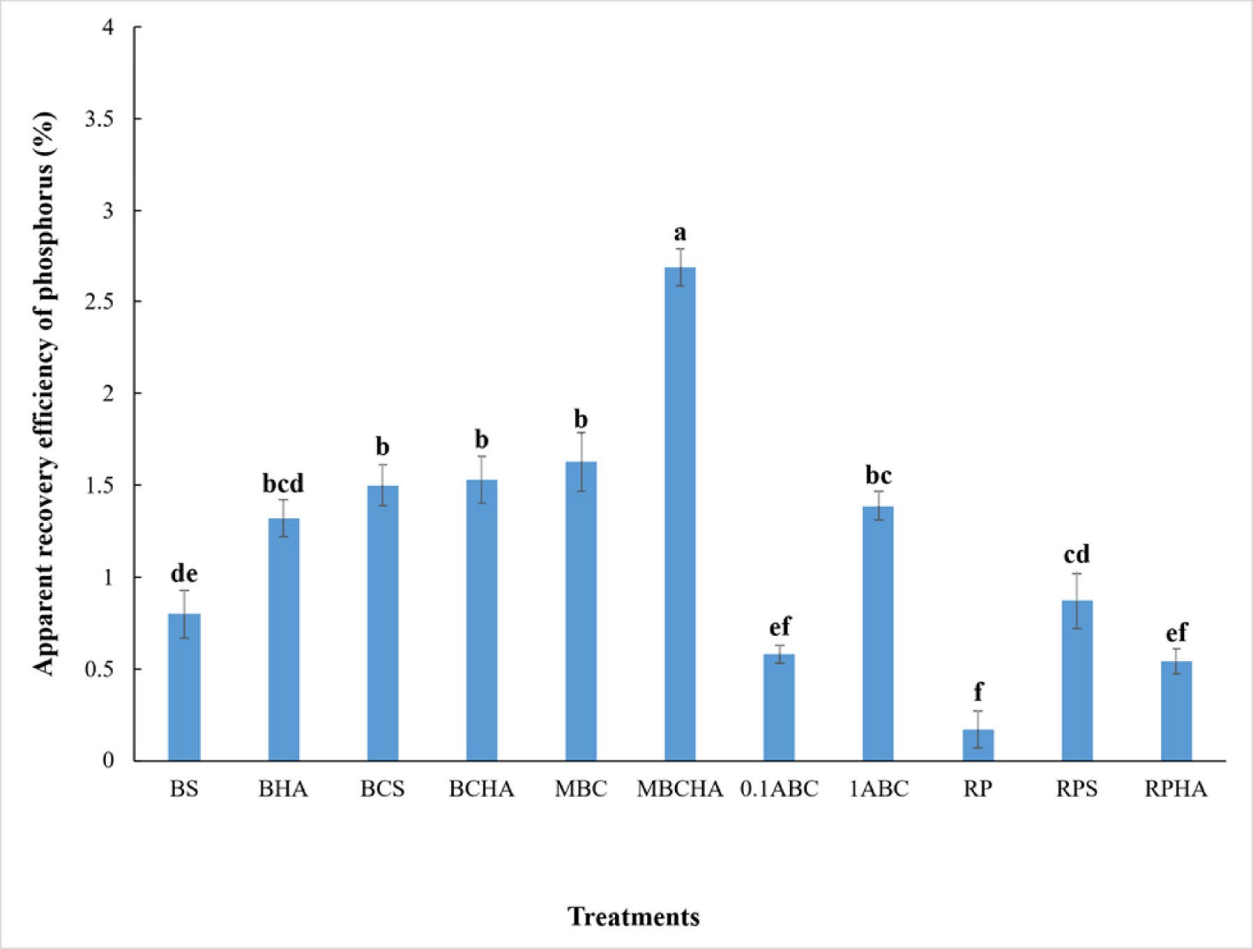
Variability in the apparent recovery efficiency of phosphorus by spinach plant grown in calcareous sandy soil as affected by different amendments. Each value indicates the average of three replicates, with the standard error shown by the vertical bars. Different lowercase letters on each bar indicate the significant differences among treatments by using Tukey’s Honestly Significant Difference test at *p* < 0.01 (corresponds to a 99% confidence level). CK: control (unamended soil); BS: bone + sulfur; BHA: bone + humic acid; BCS: bone char + sulfur; BCHA: bone char + humic acid; MBC: modified bone char; MBC + HA: modified bone char + humic acid; 0.1ABC: acidified bone char with 0.1 N H_2_SO_4_; 1ABC: acidified bone char with 1 N H_2_SO_4_; RP: rock phosphate; RPS: rock phosphate + sulfur; RPHA: rock phosphate + humic acid.

Most treatments showed significant differences in their effect on the apparent recovery efficiency of phosphorus by spinach plant (Fig. 5). The highest value of apparent recovery efficiency of phosphorus 2.69% was obtained with the application of modified bone char with humic acid, however, the lowest value of apparent recovery efficiency of phosphorus 0.17% was noticed under the application of alone rock phosphate. Also, combining bone char with humic acid or sulfur showed satisfying results for the apparent recovery efficiency of phosphorus by spinach plant grown in calcareous sandy soil. The effectiveness of the amendments under study in increasing the apparent recovery efficiency of phosphorus was in the order of MBCHA > MBC > BCHA > BCS > 1ABC > BHA > RPS > BS > 0.1ABC > RPHA > RP (Fig. 5). Applying phosphorus amendments significantly increased the phosphorus budget in the soil after harvesting spinach compared to the control treatment without phosphorus application. However, phosphorus amendments among themselves also showed significant differences in the effect on the phosphorus budget in the soil (Table 4). Phosphorus budget in control treatment without phosphorus application was 5.74 mg pot^− 1^, whereas in the presence of phosphorus fertilizers, the values of phosphorus budget were 880.33, 879.90, 873.49, 872.31, 871.26, 859.54, 880.54, 875.85, 894.48, 882.84, and 886.63 mg pot^− 1^ for BS, BHA, BCS, BCHA, MBC, MBCHA, 0.1ABC, 1ABC, RP, RPS, and RPHA, respectively (Table 4). In this study, adding phosphorus amendments resulted in the highest surpluses of phosphorus in the soil. However, the lowest surplus of phosphorus in the soil was observed in the control treatment. The effectiveness of the amendments under study in decreasing the budget of phosphorus in the order of RP > RPHA > RPS > 0.1ABC > BS > BHA > 1ABC > BCS > BCHA > MBC > MBCHA > CK (Table 4).

### Correlations between the phosphorus budget and some parameters of soil and plant

The correlation coefficients between the phosphorus budget and some parameters of soil and plant are presented in Table 6. The phosphorus budget was significantly positively correlated with available phosphorus (*r* = 0.635^**^) and phosphorus uptake (0.490^**^). The phosphorus budget was significantly negatively correlated with soil pH (*r*=-0.466^**^). The correlations between phosphorus budget with fresh weight of spinach (0.119) and dry weight of spinach (0.106) were positive and non-significant. The available phosphorus was significantly positively correlated with the fresh weight of spinach (0.364^*^), dry weight of spinach (*r* = 0.444^**^), and phosphorus uptake (*r* = 0.923^**^). The available phosphorus was significantly negatively correlated with soil pH (*r*=-0.721^**^). The soil pH was significantly negatively correlated with phosphorus uptake (*r*=-0.761^**^).

**Table 6:** The bivariate correlation test between phosphorus budget, available P, soil pH, fresh weight of spinach, dry weight of spinach, and P uptake.

	Phosphorus budget	Available phosphorus	Soil pH	Fresh weight of spinach	Dry weight of spinach	Phosphorus uptake
Phosphorus budget	1					
Available phosphorus	0.635^**^	1				
pH	-0.466^**^	-0.721^**^	1			
Fresh weight of spinach	0.119	0.364^*^	-0.007	1		
Dry weight of spinach	0.106	0.444^**^	-0.055	0.948^**^	1	
Phosphorus uptake	0.490^**^	0.923^**^	-0.761^**^	0.308	0.416^*^	1

** Correlation is significant at *p* ≤ 0.01 (corresponds to a 99% confidence level); * Correlation is significant at *p* ≤ 0.05 (corresponds to a 95% confidence level).

## Discussion

Co-applying sulfur with bone char led to a significant decrease in soil pH compared to unamended soil^16^. Several studies have found that when elemental sulfur is added to alkaline soil, the pH of the soil decreases^38,39^ as a result of oxidizing sulfur by microorganisms, which in turn leads to the production of sulfuric acid^39,40^. Humic acid plays a vital role in enhancing the physical, chemical, and biological properties of the soil as well as plant growth and agronomic parameters^41^. The application of humic acid to alkaline soil caused a significant decrease in pH^42^. Humic acid’s capability to cause changes in soil pH depends on the quantity of the functional groups, such as carboxyl and phenolic, it contains^41^. Soil pH is considered to be the “master variable” of soil chemistry because it influences the majority of chemical reactions, especially the availability of plant nutrients^43^. The co-application of sulfur and bone char in sandy soil led to a significant increase in electrical conductivity compared to the control treatment^16^. This is attributed to the oxidation of elemental sulfur producing H^+^ and SO_2_^4−^ ions, which increase the salt content of the soil^44^. The application of sulfur to sandy calcareous soil containing bone char likely increased soluble calcium as a result of the partial dissolution of biological apatite from the bone char and calcium carbonate in the soil^16^. Another reason is that sulfur in calcareous sandy soils is oxidized by sulfur-oxidizing microorganisms, lowering the pH and dissolving CaCO_3,_ which in turn improves the availability of nutrients in the soil, such as phosphorus, as well as significantly increasing soluble sulfate concentration^39^. The addition of organic amendments in combination with sulfur to calcareous soils with low organic carbon content promotes the biological oxidation of sulfur through improved microbial growth by providing labile organic carbon as an energy source to the soil microorganisms, which in turn leads to decreased pH values in these soils^45^.

Compared to unamended soil, the application of bone char significantly increased total nitrogen in the soil because the bone char contains nitrogen^46^. Also, elemental sulfur contributes to improving nutrient availability, enhances soil quality, and conserves soil health, as well as plays a vital role in causing important changes in the soil properties^47,48^. Sulfur application to the soil caused a significant increase in available nitrogen, which may be attributed to a decrease in soil pH resulting from the sulfur oxidation, which reduces the loss of nitrogen through volatilization on ammonia form^49^. The amount of phosphorus released from bone char in many soils is dependent on the components of the soil solution, organic matter content, mineralogical composition^22,23,50^, soil pH, and soil capacity to adsorb phosphorus^46^. Compared with rock phosphate, there is a higher concentration of available phosphorus in many different soils that have been amended with bone char^20,50^. The amount of plant-available phosphorus in the soil increased about seven times upon bone char application relative to unamended soil^46^. The application of sulfur with bone char significantly enhanced phosphorus availability (Olsen-P) in P-poor sandy soil compared to unamended soil^16^. An increase in the amount of phosphorus released from cow bone char was observed as a consequence of the application of elemental sulfur, which is attributed to an increase in the solubility of calcium phosphates. This is caused by the oxidation of elemental sulfur, producing H^+^ and SO_4_^2−^ ions, which decreases soil pH^44^. In calcareous soils in which sulfur is added, phosphorus release from fixation sites occurs due to sulfate ions displacing phosphorus onto the exchange sites^40^. Applying sulfur with rock phosphate (apatite) to calcareous sandy soil has successfully increased phosphorus availability. The role of sulfur in dissolving rock phosphate is due to the oxidation of sulfur to sulfuric acid, which reacts with calcium phosphate and calcium carbonate, forming calcium sulfate and releasing phosphorus from the rock phosphate^51^. Also, the presence of the sulfate anion resulted in a faster phosphorus release from nano-bone char than would have been produced by the chloride and nitrate anions^52^. Available potassium in some soils significantly increased with the applications of sulfur due to the dissolution of potassium-bearing minerals found in high quantities in calcareous sandy soil by sulfuric acid produced from the oxidation of sulfur by soil microorganisms^53^.

Generally, applying humic acid to the soil leads to a significant increase in the content of total and available nitrogen, phosphorus, and potassium^42^. Soil application of humic acid increases available nitrogen because humic acid contains nitrogen and can retain ammonium^41^. Co-application of humic acids with phosphate fertilizer in the soil significantly increased phosphorus availability^54,55^, a strong retardation of occluded phosphate formation was observed, increased P uptake, and improved wheat yield^55^. Humic acid plays an important role in increasing the availability of phosphorus in the soils through many mechanisms, such as the decrease in pH due to the decomposition of humic acid, which leads to the dissolution of phosphorus in the calcareous soils. Hydrogen ions produced from humic acids can also inhibit the precipitation of hydroxyapatite. Humic acids compete with P for sorption onto soil colloids, resulting in higher P concentrations^7^. Humic acid applications to the soil improved the efficiency of phosphate fertilizers^56^. Co-applications of humic acid with monocalcium phosphate can increase the availability of phosphorus in the calcareous soil^54^. Furthermore, interactions between the soil microbiome and crops also contribute to improving the available phosphorus in the soil^36^. The presence of humic acid may dissolve insoluble phosphates, increasing phosphorus availability by the formation of humic acid-metal-phosphate complexes, which contribute to improving soil fertility and the coupled plant growth. This is attributed to the mechanism associated with the ability of organic acids to dissolve insoluble phosphate compounds, which depends on the total number of carboxyl and hydroxyl groups to form complex metal cations, and that way weakening the phosphate precipitation equilibrium^57^. Also, the carboxyl group in humic acid may be complexed with calcium ions and phosphate ions to form a humic acid-metal-phosphate complex, which reduces the fixation of phosphorus by metal ions and increases phosphorus availability in the soil, thereby promoting plant uptake. Additionally, humic acid promotes the activity of soil microorganisms involved in phosphorus transformation, leading to increased soil phosphorus availability through enhanced phosphorus mineralization and solubilization^58^. This is attributed to the superiority of humic acid with modified bone char or bone char in increasing the availability of phosphorus in this soil. Humic acid can promote the growth and activity of sulfur-oxidizing bacteria in the soil by providing a carbon source and enhancing nutrient availability.

Bone char application to the soil can improve soil health, which in turn can enhance crop productivity and quality^59^. Applying bone char to the soil improved spinach growth, which can be attributed to increased soil nutrient availability and nutrient uptake by the spinach plant. This was proven in this study, as the concentrations of nutrients in the soil increased, such as phosphorus, calcium, and magnesium, during the growth period. These results are similar to previous studies with bone char application on many plants. Bone char effects on phosphorus availability and crop productivity showed a similar effect as chemical phosphate fertilizers on phosphorus under long-term applications, which indicates that slaughterhouse waste can be used as an alternative strategy to meet the high demand for phosphate fertilizers, especially in developing countries where phosphate reserves are limited^46^. Adding bone char to the soil led to an increase in the yield of corn and soybeans. This is due to an increase in the available amounts of phosphorus, calcium, and magnesium^46^. Bone char application significantly improved the parameters of plant growth, such as shoot weight and chlorophyll content of the corn plant. This is because bone char contains nutrients, which makes it an important organic fertilizer that helps improve fertility and enzymatic activity of the soil, thus enhancing plant growth^60^. Improving growth and grain yield of plant under the application of sulfur was due to the increasing nutrient availability, such as nitrogen, phosphorus, and potassium in the soil^49^. The addition of humic acid into the sandy loam soil improved nutrient uptake and the green yield of spinach^61^. Increasing spinach yield with the sulfur application may be due to increased availability, absorption, and transfer of sulfur to and from the spinach plant, increased enzyme activity, photosynthesis, sugar transport, and protein synthesis, as well as increased uptake of N, P, and K by the spinach crop^62^. The application of bone char led to the improvement of root and shoot growth of the plants, which will increase the plant’s ability to uptake nutrients from the soil^46^. The increased uptake of nitrogen, phosphorus, and potassium resulting from the addition of humic acid is because it plays an important role in the soil ecosystem as it provides substrates for microbial decomposition, and improves the soil’s physical, chemical, and biological properties, as well as enhances nutrient availability of nitrogen, phosphorus, and potassium^63^. Even though plant phosphorus uptake varies with soil conditions, species differences, and root development, the outcomes effectively captured the soil’s capability to release available phosphorus^64^.

The apparent recovery efficiency of phosphorus demonstrates the difference between the contributions of native soil phosphorus that is taken up in unfertilized plants compared to the fertilized plants by phosphorus^65^. The apparent recovery efficiency of phosphorus by wheat plant significantly increased with the application of sulfur in the soil is attributed to the synergistic effect between phosphorus and sulfur at low doses of phosphorus^66^. The apparent phosphorus recovery efficiency increased significantly when applying sulfur-enriched bone char compared to applying bone char^37^. Applying sulfur-enriched bone char to the soil significantly decreased the P budget compared to applying bone char. This indicates that the dissolution of phosphorus in the soil resulting from the addition of sulfur-enriched bone char is much higher than that of bone char, according to the P budget and P uptake^37^. High P budgets represent that more amount of fertilizer was applied than the plants’ uptake and that the soil contains plant-available P. This may indicate that the added P was non-soluble and non-available for plants, or that soil sorbed the dissolved P from fertilizer^37^. Some studies demonstrated that a strong positive linear relationship exists between the soil P budget and the increase in available phosphorus (Olsen-P)^36,67^. This indicates that phosphorus budgets can be a suitable tool to predict the effects of phosphorus management on plant-available P pools in soil^68^. Also, crop yield exhibited a significant positive relationship with soil available P and the P budget. The P budget drove the increase in yield significantly^36^. When a P surplus occurs within a soil, inorganic and organic P fractions from P budgets buffer the increase in dissolved P through P adsorption onto mineral surfaces, precipitation as secondary P minerals, and immobilization as microbial or organic P^69^.

Valorizing and converting bone wastes into bone char can be considered a promising and environmentally sustainable material consistent with circular economy principles^12^. The phosphorus release behavior of bone char indicates its potential as a slow-release phosphorus source, exhibiting moderate solubility compared to conventional soluble and insoluble fertilizers. Bone char has been recognized as a safe, renewable, and sustainable alternative source of phosphate fertilizer, characterized by a slower solubility rate compared to conventional soluble phosphate fertilizers^70^. Furthermore, bone char is characterized by the absence of organic contaminants, such as pharmaceuticals and heavy metals, distinguishing it from rock phosphate-based fertilizers^13,37^. The application of bone char to the soil may be a promising recycled material alternative for conventional mineral-based phosphorus fertilizers such as triple superphosphate, which would represent a move towards more sustainable agriculture with more closed phosphorus cycles^71^. Regarding environmental concerns, especially water pollution, bone char offers benefits compared to highly soluble phosphorus compounds, as the slow release of phosphorus makes it less susceptible to leaching^72^. The findings also revealed that bone char provides greater amounts of available phosphorus to plants and thus represents an effective option for supplying phosphorus in alkaline soil (high pH), performing better than phosphate rock. Moreover, this slower release of phosphorus from the bone char may be an advantage in the long term. Recycling fertilizers plays a crucial role in making the agricultural sector more sustainable and resilient.

According to the prevailing local market conditions in Assiut Governorate, the average prices of the two amendments were approximately $105.24 per ton for elemental sulfur (equivalent to about 5,000 EGP) and $3683.44 per ton for humic acid (equivalent to about 175,000 EGP), indicating a wide disparity in their market values and potential economic implications for agricultural use. Economically, the application of elemental sulfur represents a much more affordable alternative to humic acid, as its market price is only a small fraction of that of humic acid, making it a more feasible option for large-scale agricultural practices. Therefore, the combination of bone char with sulfur is economically preferable owing to the relatively low cost of sulfur compared to humic acid.

Generally, the limitations facing bone char applications as fertilizer in the soils include: (1) the low solubility of phosphorus compounds in bone char, (2) soil characteristics play a pivotal role in influencing phosphorus solubility from bone char, (3) bone char preparation, (4) single crop and short cultivation duration, and (4) carried out field experiments on the use of bone char as a fertilizer in soil are very limited. Despite all these limitations, the high phosphorus content of bone char is considered a potential source of phosphorus, and future studies should focus on long-term field experiments across different soil types, crops, and climatic conditions to validate these findings. Further investigation into the interaction of bone char with soil microbes and other amendments is also recommended to enhance phosphorus availability and overall nutrient efficiency, as well as environmental assessments.

## Conclusion

Implementing circular economy principles represents a key opportunity for fertilizer production to achieve sustainability, minimize waste generation, and enhance agricultural productivity. Innovative recycling and resource recovery practices are essential steps toward securing the future of sustainable agriculture. The high price and environmental problems associated with the continuous use of phosphate fertilizers represent major challenges in modern agriculture. We must find alternatives to phosphate fertilizers that are cheap, clean, and safe. This, in turn, is consistent with sustainable agricultural development. The goals of this study were to examine the influence of applying modified bone char as well as co-applying bone char with sulfur and humic acid on soil chemical properties, phosphorus availability, and growth of spinach in calcareous sandy soil. The results of this study showed that the effectiveness of the treatments in this study on enhancing available phosphorus was in the order of modified bone char + humic acid > modified bone char > bone char + humic acid > bone char + sulfur > acidified bone char with 1 N H_2_SO_4_ > bone + humic acid > bone + sulfur > acidified bone char with 0.1 N H_2_SO_4_ > rock phosphate + sulfur > rock phosphate + humic acid > rock phosphate > control. In this study, bone char and bone treatments were better than phosphate rock treatments in improving phosphorus availability in calcareous sandy soil. Effectiveness of treatments in improving the fresh weight of spinach was in the order of BCHA > BCS = BHA > RPS > MBCHA > MBC > RPHA > 1ABC > control > RP > BS > 0.1ABC. Co-applying bone char with sulfur and humic acid treatments is one of the best treatments that led to improving the fresh weight of spinach, but co-applying bone char with sulfur is preferred due to the cheap price of sulfur. In sustainable agriculture, the use of bone char with sulfur is considered a promising strategy in confronting the shortage of phosphate fertilizers, as it is a cheap and clean alternative to phosphate fertilizers. This work is a preliminary study that provides a proof-of-concept demonstrating the potential of bone char combined with sulfur as a sustainable phosphorus source. It is recommended to conduct further research and field experiments to select the appropriate doses of bone char application and evaluate its long-term effects on crop productivity in the soils and environmental assessments.

## Data Availability

The datasets used or analyzed during the current study are available from the corresponding author upon reasonable request.

## Abbreviations

0.1ABC: Acidified BC with 0.1 N H_2_SO_4_
1ABC: Aacidified BC with 1 N H_2_SO_4_
B: Bone
BHA: Bone + humic acid
BS: Bone + sulfur
BC: Bone char
BCHA: Bone char + humic acid
BCS: Bone char + sulfur
C: Carbon
Ca: Calcium
CaCO_3_: Calcium carbonate
CK: Control
Cl: Chloride
EC: Electrical conductivity
H: Hydrogen
H_2_O_2_: Hydrogen peroxide
H_2_SO_4_: Sulfuric Acid
ha: Hectare
HA: Humic acid
HCl: Hydrochloric acid
HCO_3_: Bicarbonate
K: Potassium
MBC: Modified bone char
MBCHA: Modified bone char + humic acid
Mg: Magnesium
N: Nitrogen
Na: Sodium
NaOH: Sodium hydroxide
O.M: Organic matter
P: Phosphorus
p: Probability
RP: Rock phosphate
RPHA: Rock phosphate + humic acid
RPS: Rock phosphate + sulfur
S: Sulfur
SO_4_: Sulfate
